# Effect of Malondialdehyde-Induced Oxidation Modification on Physicochemical Changes and Gel Characteristics of Duck Myofibrillar Proteins

**DOI:** 10.3390/gels8100633

**Published:** 2022-10-06

**Authors:** Xueshen Zhu, Zhenghao Ma, Xinyu Zhang, Xuefang Huang, Junya Liu, Xinbo Zhuang

**Affiliations:** 1 Key Lab of Biological Functional Molecules of Jiangsu Province, College of Life Science and Chemistry, Jiangsu Second Normal University, Nanjing 211200, China; 2 College of Food Science and Engineering, Nanjing University of Finance & Economics, Nanjing 211171, China

**Keywords:** malondialdehyde, duck meat, myofibrillar proteins, physicochemical changes, gel properties

## Abstract

This paper focuses on the effect of malondialdehyde-induced oxidative modification (MiOM) on the gel properties of duck myofibrillar proteins (DMPs). DMPs were first prepared and treated with oxidative modification at different concentrations of malondialdehyde (0, 0.5, 2.5, 5.0, and 10.0 mmol/L). The physicochemical changes (carbonyl content and free thiol content) and gel properties (gel whiteness, gel strength, water holding capacity, rheological properties, and microstructural properties) were then investigated. The results showed that the content of protein carbonyl content increased with increasing MDA oxidation (*p* < 0.05), while the free thiol content decreased significantly (*p* < 0.05). Meanwhile, there was a significant decrease in gel whiteness; the gel strength and water-holding capacity of protein gels increased significantly under a low oxidation concentration of MDA (0–5 mmol/L); however, the gel strength decreased under a high oxidation concentration (10 mmol/L) compared with other groups (0.5–5 mmol/L). The storage modulus and loss modulus of oxidized DMPs also increased with increasing concentrations at a low concentration of MDA (0–5 mmol/L); moreover, microstructural analysis confirmed that the gels oxidized at low concentrations (0.5–5 mmol/L) were more compact and homogeneous in terms of pore size compared to the high concentration or blank group. In conclusion, moderate oxidation of malondialdehyde was beneficial to improve the gel properties of duck; however, excessive oxidation was detrimental to the formation of dense structured gels.

## 1. Introduction

In general, myofibrillar proteins are important biological function proteins. The content of myosin and actin exceeds 2/3 of them. Myofibrillar proteins have better functionality and changes in their structure lead to changes that produce good textural characteristics, whose gel characteristics are the basis of processed minced meat products and directly affect the sensory properties of the final meat product [1]. In particular, oxidative modifications show the most pronounced effects. Oxidation of proteins can weaken the protein interactions and thus alter their gel formation, which has an impact on the quality of the cleavage, and intermolecular interactions lead to secondary/tertiary structural changes in the protein [2]. Side chains contain amino acids such as arginine, tyrosine, leucine, cysteine, phenylalanine, histidine, tryptophan, proline, lysine, and methionine, which are very sensitive to the action of reactive oxygen species (ROS) [3]. Among them, histidine, being highly sensitive to sulfide centers, could be oxidized at lower ROS concentrations, while leucine and proline are converted to hydroxyl derivatives. In addition, lysine is more likely to form carbonyl residues under metal catalysis [4]. Sulfur-containing amino acids such as cysteine are most susceptible to oxidation by free radicals, which is reversible, and it has some cyclic oxidation and reduction [5].

It is known that malondialdehyde (MDA) is naturally produced under meat processing conditions and is the most abundant individual aldehyde produced by lipid peroxidation. At certain ionic strengths, MDA can further promote gelation of myofibrillar proteins [6]. Zhou et al. [7] found that gels are formed by the solubilization of myofibrillar proteins at certain ionic strengths and form nondisulfide covalent bonds with MDA, a secondary fat oxidation product. Previous studies have also confirmed that the secondary and tertiary structures of myofibrillar proteins are readily altered by oxidation, leading to the unfolding of myofibrillar protein structures, which further promotes effective protein-protein interactions [8]. Thus, the oxidation of protein structures leads to changes in their physical properties, which are related to the degree of oxidation of the protein, and in general, the longer the oxidation time, the worse the functional properties of the protein [9]. It is also important to mention that, according to Wang et al. [10], mild oxidation facilitates the formation of an elastic gel lattice structure, while excessive oxidation decreases the copolymerization force and elasticity of the gel. MDA can also bind to proteins, altering protein interactions and further leading to changes in the functional properties of myofibrillar proteins in processed muscle foods. Xiong et al. [11] concluded that MDA can change the conformation of myofibrillar proteins by altering their side chains and polypeptide backbone. In addition, MDA can react with the amino group of myofibrillar proprotein to produce a strong Schiff-base-type intermolecular cross-linking, which also promotes gel formation [12]. However, few studies have been conducted on the mechanism of action of MDA on duck myofibrillar proteins (DMPs), which has been selected as an effective secondary oxidation product during lipid metabolism. Therefore, we hypothesized that direct incubation of MDA with DMPs could alter the structure of DMPs, leading to affect the formation of compact and rigid gels. The aim of this study was to investigate the effect of oxidative modification of malondialdehyde on the physicochemical changes and gel properties of DMPs, aiming to provide a basis for controlling the degree of meat oxidation and the rational use of oxidants.

## 2. Results and Discussion

### 2.1. Morphological Observation

**Figure 1:**
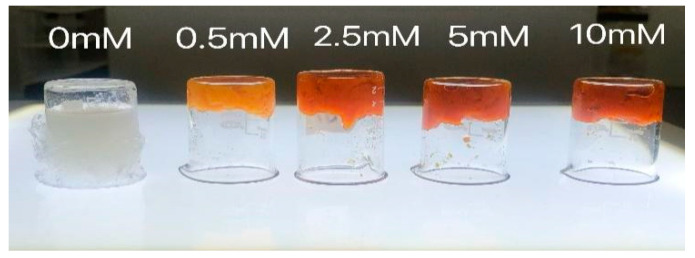
Morphology observation of duck myofibrillar proteins gels treated with different malondialdehyde concentrations (0, 0.5, 2.5, 5.0, and 10.0 mmol/L).

Figure 1 shows the results of malondialdehyde-induced oxidation modification on the gel morphology of duck myofibrillar proteins (DMPs). As shown in Figure 1, the gel morphological state of the DMP samples (dissolved in 0.6 M NaCl) changed from solution to gel as the MDA concentration increased. It also clearly showed that gel samples treated at higher concentrations of MDA (0.5, 2.5, 5.0, 10 mM) became brown and gel-like and did not collapse easily, while the blank group without MDA remained sol-like and flowed easily.

### 2.2. Carbonyl Content and Free Thiol Content Changes

**Figure 2:**
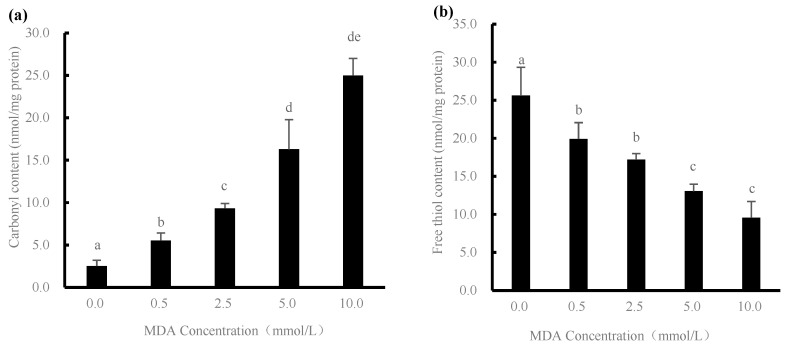
Changes in carbonyl content (**a**) and free thiol content (**b**) of duck myofibrillar proteins treated with different malondialdehyde (MDA) concentrations (0, 0.5, 2.5, 5.0, and 10.0 mmol/L). Different letters (a–e) indicate significant difference (*p* < 0.05).

Protein carbonylation is an important oxidation reaction of proteins that leads to the oxidation of new carbonyl groups due to their ‒NH‒ or ‒NH_2_ groups on the side chains of amino acids [4]. Figure 2a shows the changes in the carbonyl content of the samples after oxidative treatment with different concentrations of MDA. The protein carbonyl content of the samples treated in the absence of MDA was 2.52 nmol/mg protein. The carbonyl content of DMPs increased significantly (*p* < 0.05) with increasing MDA concentration from 0 to 10 mmol/L, because MDA could be added to primary amines in protein molecules in a certain ratio to produce enamine adducts, resulting in new carbonyl groups. In addition, the molecular structure of MDA is composed of two carbonyl groups combined, where the oxidation of one MDA molecule with the protein can introduce another carbonyl group at the same time. In general, MDA could act on the nucleophilic side chain groups of cysteine, histidine, and lysine residues, which then react to form the Schiff base [13].

In proteins, sulfhydryl groups are much reactive, so sulfur-containing amino acid residues can easily interact with each other to produce disulfide bonds, and the earliest oxidation of sulfhydryl groups in proteins can lead to changes in protein structure, which, in turn, can have some effects on protein function [14]. As shown in Figure 2b, the free thiol content of DMPs decreased gradually from 25.62 to 9.58 nmol/mg of protein with increasing MDA concentration, which was due to the exposure of endogenous sulfhydryl groups during the breakdown of the protein structure and exposure to pro-oxidant compounds, resulting in a constant decrease in the total amount of free thiol groups, as confirmed by the recent findings of Soglia et al. [15]. Previous reports have also indicated that the loss of sulfhydryl groups in plasma proteins occurs due to the interaction between the sulfhydryl groups of proteins and the α, β‒Michael addition of unsaturated aldehydes [16]. In addition, MDA is electrophilic and can preferentially abstract hydrogen atoms from the free thiol group of cysteine, leading to a decrease in free thiol content [17].

### 2.3. SDS-PAGE Profile Analysis

**Figure 3:**
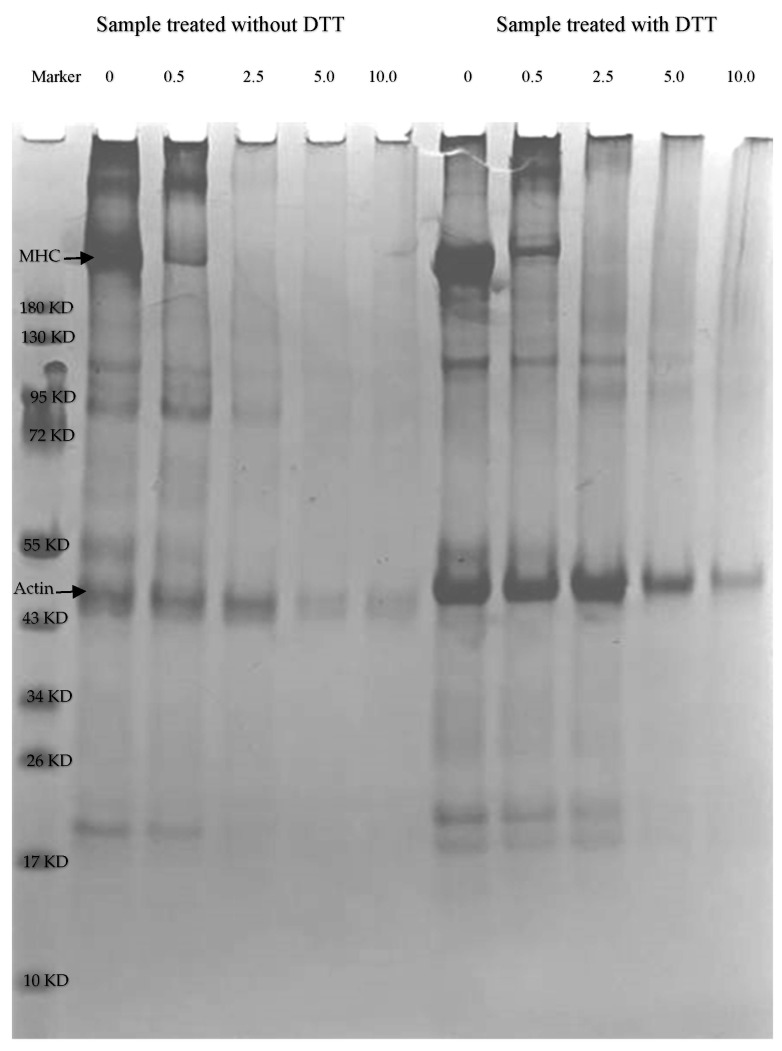
SDS-PAGE pattern changes of duck myofibrillar proteins treated with different malondialdehyde concentrations (0, 0.5, 2.5, 5.0, and 10.0 mmol/L).

As shown in Figure 3, the intensity of myosin heavy chain (MHC) bands was largely diminished in samples under nonreducing conditions (without Dithiothreitol, ‒DTT) after treatment with 2.5, 5.0, and 10.0 mmol/L MDA, respectively. Other bands of myofibrillar proteins disappeared (actin bands were slightly blurred) when the MDA concentration exceeded 2.5 mmol/L, a phenomenon indicating that with increasing oxidation concentration, protein cross-linking overloaded. Under reducing conditions (with dithiothreitol, +DDT), similar results were found as under nonreducing conditions (‒DTT), where the disappeared myosin heavy chain components were not recovered and the large polymer remained in the loaded part of the gel, indicating that the cross-linking was not essentially through disulfide bonds or similar mechanisms and that the covalent bonds of this cross-linking were strong and difficult to break. It is important to mention here that fewer polymers were formed under nonreducing conditions compared to reducing conditions. We supposed that this might be due to the excessive cross-linking of proteins caused by high concentrations of MDA, forming polymers of very high molecular weight that simply cannot enter the separating gel [18]. Riley and Harding [19] reported that malondialdehyde can react with lens proteins to form covalent cross-linkages with nondisulfide bonds. Oxidative modification at different concentrations of MDA with 0.6 mmol/L NaCl resulted in the unfolding of myosin, which increased action sites, and also the formation of non-disulfide bonds was not conducive to separation between myosin and related proteins by SDS-PAGE. In the presence of MDA, the cross-linking of proteins caused by nondisulfide bonds is more likely to be associated with the formation of Schiff bases as described above. Recent findings also suggest that protein aggregation occurs in the MDA-induced oxidation system. Myosin is involved in gel formation through nondisulfide covalent bonds [20]. Disulfide bonds are responsible for most of the cross-linking and malondialdehyde also seems to contribute to cross-linking [11].

### 2.4. Gel Strength and Water Holding Capacity Analysis

**Figure 4:**
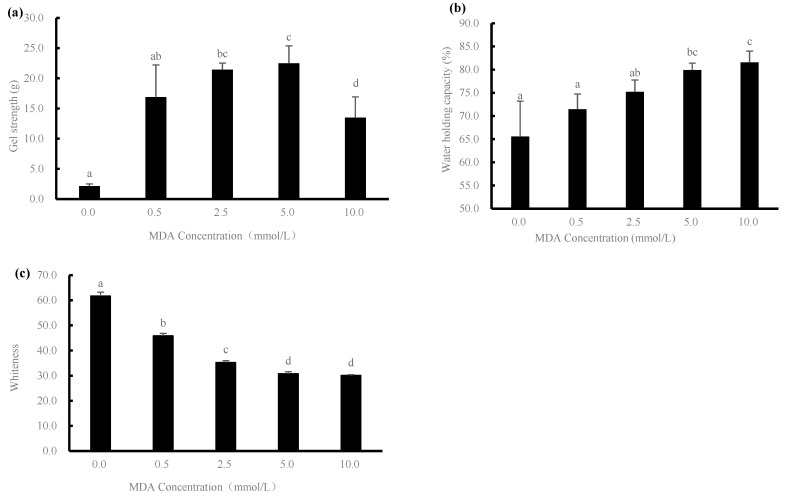
Changes in gel strength (**a**), water holding capacity (**b**), and whiteness (**c**) of duck myofibrillar proteins treated with different malondialdehyde (MDA) concentrations (0, 0.5, 2.5, 5.0, and 10.0 mmol/L). Different letters (a–d) indicate significant difference (*p* < 0.05).

Gel strength is an important quality characteristic of protein gels. As shown in Figure 4a, the oxidative modification induced by MDA played an important role in affecting the gel strength. It is clearly shown that the gel strength of the sample increased significantly from 0.02 N to 0.23 N with increasing MDA concentration. This could be due to the creation of more cross-linking among proteins through nondisulfide bonds and disulfide bonds during the thermal treatment of the gel, and the formation of these covalent bonds promoted the strengthening of the gel structure; thus, the gel strength of the sample was improved [21]. However, when comparing the 5 mmol/L treatment group with the 10 mmol/L treatment group, gel strength decreased significantly with the further increase in MDA concentration (*p* < 0.05). The reason could probably be due to the oxidation at high concentrations leading to the formation of excessive covalent bonds, which, in turn, might have promoted the degradation of proteins and decrease in gel strength [22]. Previous studies have shown that disulfide and other covalent bonds are enhanced, leading to protein cross-linking and aggregation, which affects gel strength, while enhancing the degradation of actin and troponin-T [23].

Similar to gel strength, analysis of the water holding capacity of gels can also reflect the quality of differently treated myofibrillar protein gels. The water holding capacity of a gel is related to the spatial structure of the gel and is a response to the ability of the gel to retain water molecules [24]. As shown in Figure 4b, the water holding capacity (WHC) of the gel samples increased continuously with the increase of MDA concentration (0–10 mmol/L), from 65.6% initially to 85.55%, and the trend of its water holding capacity was basically consistent with that of the carbonyl group. It could be speculated that MDA promoted the carbonylation of proteins and was responsible for the formation of protein crosslinks throughout the incubation treatment, thus changing the reticular structure of the gels and promoting the improvement of the water holding capacity of the gels [25].

As shown in Figure 4c, whiteness decreased significantly with increasing MDA content, and the results indicate that the brightness, redness, and yellowness values of the gels were significantly affected, which corresponds to the morphological observations described previously. Xia et al. [26] also found that repeated thawing and freezing treatments of meat caused nonenzymatic oxidation reactions between fat oxidation products and amino acids in proteins, leading to a decrease in their whiteness.

### 2.5. Rheological Characterization

**Figure 5:**
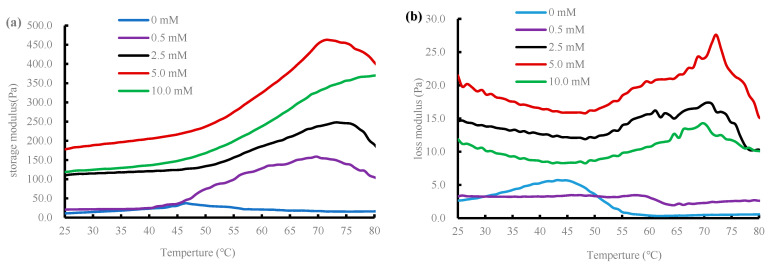
Storage modulus (**a**) and loss modulus (**b**) changes during heating of duck myofibrillar proteins treated with different malondialdehyde concentrations (0, 0.5, 2.5, 5.0, and 10.0 mmol/L).

Storage modulus is an important parameter during the determination of dynamic rheological properties, which is shown in Figure 5a. First, when the MDA concentration was 0, the curve peaked from 25 °C to 46.5 °C; afterward, the curve gradually decreased until 70.1 °C, and then the curve continued to increase until the end of the test, indicating that 46.5 °C was the gel point of myofibrillar protein gel. In the temperature interval from 25 to 46.5 °C, DMPs underwent high-temperature denaturation, which exposed its amino acids and led to changes in the gel structure, resulting in the formation of a gel with high elasticity; in the temperature interval from 46.5 to 70.1 °C, the original gel matrix was destroyed, and in the interval from 70.1 to 80 °C, the ordered cross-linking of disulfide bonds continued, resulting in the formation of a stable, uniform, dense, and three-dimensional elastic network with high energy density. At low MDA concentrations (0–5 mmol/L), the storage modulus trends were similar among different MiOM groups, but the final storage modulus increased significantly with increasing MDA oxidation concentrations. It should be emphasized here that the storage modulus of the 10 mmol/L treatment group was lower when compared with the 5 mmol/L treatment group. This confirmed that when treated at relatively lower oxidation concentrations of MDA, protein–protein interactions were enhanced, producing better binding ability among the proteins, whereas excessive oxidation concentrations of MDA oxidation could lead to ultra-aggregation and shrinkage of the protein gel, which was detrimental to the gel-forming ability of DMPs, as described by the previous results on gel strength of the DMPs.

The trend of the loss modulus (Figure 5b) was roughly similar with changes in the storage modulus mentioned above. The loss modulus curve (0 mmol/L) first peaked at 43.1 °C and then gradually decreased and leveled off at 56 °C. Figure 5b also clearly shows a gradual increase in the loss modulus of samples treated at different MDA concentrations (0.5–5 mmol/L) until about 70 °C, decreasing afterward, which might be due to the destruction of the previously formed gel matrix with increasing temperature [27]. In addition, the curves of the loss modulus under 10 mmol/L MDA oxidation were generally lower when compared with those of 2.5 mmol/L and 5 mmol/L treatment groups. These results coincided with the previous results of gel strength.

### 2.6. Nuclear Magnetic Characterization

**Figure 6:**
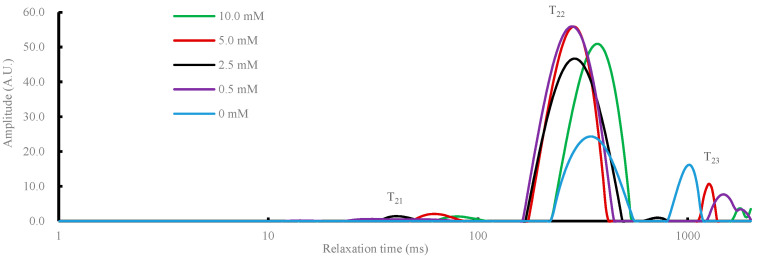
Effect of different malondialdehyde concentrations (0, 0.5, 2.5, 5.0, and 10.0 mmol/L) on the distribution of the T_2_ of duck myofibrillar proteins gel. T_2_ = spin–spin relaxation times (ms) for different types of water.

Figure 6 shows the effect of malondialdehyde concentration on the changes in relaxation time (T_2_) of the gels of DMPs. As shown in the figure, three typical peaks were found, with one peak appearing at 20–96 ms, a larger peak at 131–446 ms, and another peak after 800 ms. Our previous paper found that DMP gels generally show three peaks in the fitted NMR relaxation curves, corresponding to moderately immobilized water, immobilized water, and free water, respectively [28]. That is, T_21_ represents moderate immobilized water with peaks between 20 and 96 ms, T_22_ represents immobilized water with peaks between 131 and 446 ms, and T_23_ represents free water with peaks after 800 ms. In general, DMPs cross-link with each other to form a three-dimensional structure, locking up a large number of water molecules and forming immobilized water, which is understandable. Our results also showed that the area of T_22_ (immobilized water) increased with increasing MDA concentration (from 0 to 5.0 mmol/L). In addition, a decreasing trend of peak area could be found for the 10 mmol/L treatment group when compared with 2.5 and 5 mmol/L treatment groups. In addition, there was a decreasing trend of T_23_ (free water) with increasing MDA concentration, suggesting that especially low concentrations of MDA help to convert free water to immobilized water and reduce the free water content. These results fit well with the previous discussed results in water holding capacity, and correspond to the finding of Wang et al. [29] that the fraction of free water decreased from 7.66% to 0.15% with increasing MDA addition from 0 to 50 mM. Furthermore, the relaxation component (bound water) disappeared with the addition of MDA, mainly due to the increase in flexibility and surface hydrophobicity of the protein. Xia et al. [30] also reported that WHC had a significant positive correlation with the percentage of immobile water and a negative correlation with carbonyl content and T_23_.

### 2.7. Gel Microstructure Analysis

**Figure 7:**
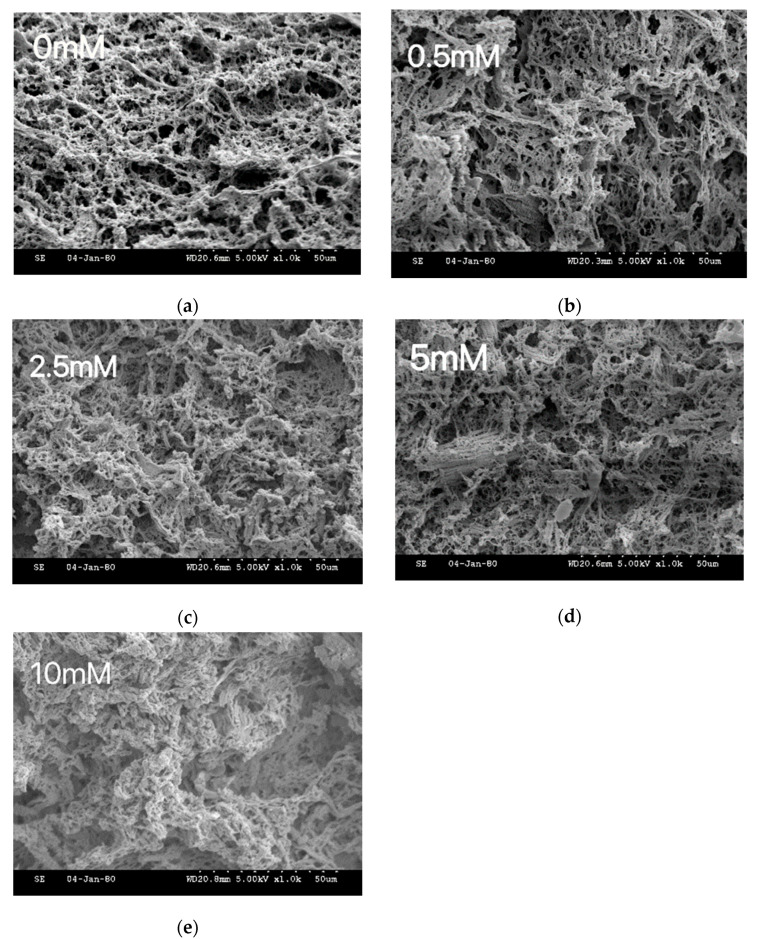
Gel microstructure analysis of duck myofibrillar proteins treated with malondialdehyde (MDA) concentrations of 0 mmol/L (**a**), 0.5 mmol/L (**b**), 2.5 mmol/L (**c**), 5.0 mmol/L (**d**), and 10.0 mmol/L (**e**).

The results of the gel microstructure analysis are shown in Figure 7. The samples treated with relatively low MDA concentrations (2.5 and 5 mmol/L) showed a better gel mesh structure, which were gradually tightened to form an ordered structure with uniform pore size. This result was consistent with the enhancement of gel strength as described previously. In contrast, the gel structure remained firm after 10 mmol/L MDA treatment, and the depression of the gel surface structure could be easily found in the figure, which could be explained by the weakening of the gel strength in our previous results. These results clearly indicated that the mesh structure of the gels could be improved at relatively mild MDA oxidation [31]. Similar results were reported by Zhou et al. [32]. Higher MDA could cause the collapse of the gel due to the presence of excessive covalent bonds.

## 3. Conclusions

MDA-induced oxidation can change the physicochemical structure of DMPs, such as a significant increase in carbonyl content and a significant decrease in free thiol content; in addition, gel whiteness and WHC showed a decreasing trend. It should be mentioned that under proper oxidation conditions, the protein gel hardness increased significantly at low concentrations of MDA (from 0 to 5 mmol/L); however, it decreased at high concentrations (10 mmol/L). The enhancement of covalent bonds promoted the consolidation of the gel structure. These results suggest that covalent bonds induced during heating at mild-low concentrations of MDA oxidation might improve the gel structure and, thus, improve the gel quality.

## 4. Materials and Methods

### 4.1. Samples and DMPs Preparation

The duck breast meat used in this experiment was obtained from the local market in Lishui, Nanjing, China. All fat was stripped off and muscles were then placed in a self-sealing bag in a −80 °C refrigerator.

To extract DMPs, 30 g of duck breast meat was added to the extract (5 times volume), homogenized at high speed for 20 s, and then placed in a refrigerated centrifuge tube and centrifuged under the following conditions: 4000× *g* at 4 °C for 10 min, repeated three times. It was then filtered through gauze, and 1% TritonX-100 (5 times volume) was used afterward to wash the above precipitate. This was repeated three times together with centrifugation methods, and it was finally washed with 0.1 mmol/L NaCl (5 times volume) to dissolve, homogenize, and centrifuge again. The supernatant was discarded and the precipitated DMPs were finally collected by filtration through gauze.

### 4.2. MDA Oxidation-Modified Myofibrillar Protein Treatment

The MDA stock solution was mainly prepared through 1,1,3,3-tetramethoxypropane obtained from Sigma-Aldrich Chemical Co. (St Louis, MO, USA) as described by Wu et al. [33] with minor modifications.

The MDA-induced oxidation system of DMPs was referred to Zhou et al. [7] with appropriate modifications. At first, the concentration of DMPs was adjusted to 40 mg/mL with MDA stock solution at different ratios (MDA concentration: 0, 0.5, 2.5, 5.0, and 10.0 mmol/L). The mixture was then placed in a 10 mL centrifuge tube and incubated at 25 °C for 24 h. The protein gels were then prepared as follows: protein solutions were heated directly in a water bath after the above treatment, subject to linear heating at a starting temperature of 25 °C to 85 °C at a speed of 1 °C/min, maintained at 85 °C for 5 min, and then cooled with an ice bath and stored at 4 °C before use.

### 4.3. Carbonyl Content Determination

The protein carbonyl content was modified according to Soglia et al. [34]. The obtained samples of DMPs were adjusted to a concentration of 20 mg/mL. During the assay, 5% SDS was used to resolve the precipitates and dinitrophenylhydrazine (DNPH) was used to label the carbonyl groups. Finally, the absorbance at 280 nm and 370 nm was measured in solution. The carbonyl content was calculated using 22,000 L/(mol·cm) as the molar extinction coefficient for conversion and expressed in units of nmol/mg protein, as shown in the equation:

(1) $$Carbonyl\;content\;\left(nmol/mg\;protein\right)=\frac{\left[\;A_{370\;}-A_{370\;\left(blank\right)}\right]\times 10^{6}}{22,000\times \left[\;A_{280\;}-\left(A_{370}-A_{370\left(blank\right)}\right)\times 0.43\right]}$$

### 4.4. Free Thiol Content Determination

The protein free thiol content was appropriately determined according to the method of Bao et al. [35]. The concentration of the oxidized DMPs obtained was adjusted to 2 mg/mL, and 300 μL of this protein concentration solution was used. Briefly, 0.5 mL of 10 mM 5,5′-dithiobis-(2-nitrobenzoic acid) was added to the solution, mixed well, and protected from light for 30 min. The molar extinction coefficient was 14,150 L/(mol·cm), and the free thiol content was calculated as follows:

(2) $$Free\;thiols\;content\;\left(nmol/mg\;protein\right)=\frac{\left[\;A_{412\left(after\right)}-A_{412\;\left(before\right)\;}\right]}{A_{280}\times 14,150}$$

### 4.5. Sodium Dodecyl Sulfate-Polyacrylamide Gel Electrophoresis Analysis (SDS-PAGE)

DMPs with different MDA-induced oxidation modification (MiOM) were adjusted to 2 mg/mL. The DMPs were pretreated using sample buffer (Invitrogen, Thermal Fisher, Waltham, MA, USA) with or without dithiothreitol (DTT, Beyotime, Shanghai, China), and then heated in a metal bath at 99 °C for 5 min. Electrophoresis was then run at 220 V for 45 min. The Coomassie Brilliant Blue R-250 (Beyotime, Shanghai, China) solution was used to stain for 30 min and then decolorized and photoed for analysis.

### 4.6. Gel Hardness and Water Holding Capacity (WHC)

The gel strength of DPM samples was determined according Zhu et al. [36] using a texture analyzer (TA-XT plus Plaser, Stable Micro Systems, Surrey, United Kingdom). Measurement conditions: P/0.5R probe with a pre-measurement speed of 1 mm/s, a measurement speed of 0.5 mm/s, a post-measurement speed of 10 mm/s, compression mode, and a depth distance of 5 mm. Each treated sample was repeated 3 times.

The WHC of the gel was determined using centrifugation methods. The gel was centrifuged at 6000 r/min for 15 min at 4 °C. The centrifuge tube was then placed upside-down on absorbent paper for 30 min. Before centrifugation, the mass of the tube was *m*, the total mass was recorded as *m*1, and the total mass after being placed for 30 min was *m*2. Water holding capacity was calculated as follows:

(3) $$WHC\;\left(\%\right)=\frac{m2-m}{m1-m}\times 100\;$$

### 4.7. Gel Whiteness Determination

The gel samples were determined using a CR 400 colorimeter (Minolta Camera, Osaka, Japan). Brightness (L*), red (a*), and yellow (b*) were measured three times for each sample, and the gel whiteness was calculated as follows:

(4) $$Whiteness=100-\sqrt{\;{\left(100-L^{*}\right)}^{2}+a^{*}{}^{2}+b^{*}{}^{2}}$$

### 4.8. Rheological Properties Test

The freshly oxidized protein solution was measured using a rheometer (MCR-301, Anton Paar, Graz, Austria) in oscillatory mode as described by Zhuang et al. [37]. The following parameters were used: A 50 mm plate material was selected with a gap of 1 mm between the upper and lower plates, a frequency of 0.1 Hz, a strain of 2%, 25 °C/min, the heating temperature was from 25 °C to 80 °C at a speed of 2 °C/min, and the cooling rate was 5 °C/min. Before the test, paraffin oil was used to drip into the edge of the plate to isolate the sample from the outside air. The storage modulus and loss modulus during heating was then recorded.

### 4.9. Low-Field Nuclear Magnetic Resonance Analysis

Relaxation times (T_2_) were measured according Han et al. [38] with a nuclear magnetic resonance (NMR) analyzer (MesoMR23-060H-1, Niumag elctric Co., Shanghai, China). A standard oil sample was first calibrated, a centrifuge tube of about 2 g was placed in the tester, and the spin–spin relaxation time (T_2_) was selected as the Carr–Purcell–Meiboom–Gill (CPMG) sequence. The proton resonance frequency was set at 22.6 MHz and the measurement was performed at a temperature of 32 °C. The relevant parameters: the number of repetition sampling (NS) was 4 times, the repetition interval (time wait, TW) was 2000 ms, the number of echoes (NECH) was 9000, each test was performed 3 times, and the obtained curve was an exponential decay sample curve. A large number of data inversions were achieved through the data query function in the software menu.

### 4.10. Gel Microstructure Analysis

The gel samples were cut into squares (3 mm × 3 mm × 3 mm) and fixed with 4% malondialdehyde. Gels were then analyzed with a Hitachi S-3000N scanning electron microscope (Tokyo, Japan) at an accelerating voltage of 20 kV.

### 4.11. Statistical Analysis

Data were processed with SPSS 20.0 software (version 20, SPSS Inc., Chicago, IL, USA) and subjected to one-way ANOVA with Duncan’s multiple range test for statistical analysis.

## Data Availability

The datasets generated for this study are available on request to the corresponding author.
